# Unveiling the hidden function of long non-coding RNA by identifying its major partner-protein

**DOI:** 10.1186/s13578-015-0050-x

**Published:** 2015-10-22

**Authors:** Yongfang Yang, Liwei Wen, Hongliang Zhu

**Affiliations:** Department of Food Biotechnology, College of Food Science and Nutritional Engineering, China Agricultural University, 100083 Beijing, China

**Keywords:** Long non-coding RNA, Function, Interaction, Protein, Biochemistry approaches

## Abstract

Tens of thousands of long non-coding RNAs (lncRNAs) have been discovered in eukarya, but their functions are largely unknown. Fortunately, lncRNA–protein interactions may offer details of how lncRNAs play important roles in various biological processes, thus identifying proteins associated with lncRNA is critical. Here we review progress of molecular archetypes that lncRNAs execute as guides, scaffolds, or decoys for protein, focusing on advantages, shortcomings and applications of various conventional and emerging technologies to probe lncRNAs and protein interactions, including protein-centric biochemistry approaches such as nRIP and CLIP, and RNA-centric biochemistry approaches such as ChIRP, CHART and RAP. Overall, this review provides strategies for probing interactions between lncRNAs and protein.

## Background

Recently, an explosion of microarray tiling and high-throughput deep sequencing analysis has led to the discovery of thousands of previously presumed non-coding transcripts [1, 2]. Global transcriptional analyses of the human genome have revealed that non-coding RNA far exceed the protein-coding mRNAs which account for only about two percent of the human genome [3]. Non-coding RNA include many small regulatory RNAs and tens of thousands of polyadenylated and nonpolyadenylated lncRNAs which have been shown to be essential for many rapidly growing research areas [4]. Although only a few lncRNA have been documented to have important biological functions, increasing evidences suggest that the regulation of lncRNAs on target genes is complicated [5].

lncRNAs, through interactions with protein, DNA and RNA, regulate gene expression at multiple levels, including chromatin remodeling and nuclear transcription, pre-mRNA splicing and cytoplasmic mRNA translation [6]. Moreover, virtually all functional RNA molecules interact with protein complexes and protein is confirmed to be the first and principal partner of lncRNA [7]. Thus, understanding lncRNA functions can be accomplished by identification of lncRNA-bound proteomes. Substantial effort is being devoted to depicting RNA–protein interactions for gaining insight into molecular mechanisms, but lncRNA–protein interplay is poorly understood [8–10]. In this review, we highlight molecular modes and functions of lncRNA–protein interactions and summarize conventional and emerging techniques to probe these interactions, with hopes of illuminating hidden lncRNA regulatory mechanisms.

## Characteristics and function of lncRNA

LncRNA are a group of non-coding RNAs defined as being larger than 200 nucleotides in length, which distinguish them from small RNAs such as microRNAs, small nucleolar RNAs (snoRNAs) and small interfering RNAs (siRNAs) [11]. According to the relative position of the coding gene, lncRNA exist in four groups: intergenic, introngenic, overlap and antisense [12]. Compared with protein coding RNA, lncRNAs are typically shorter with fewer exons, less abundance, less coding potential and more restrictions to particular tissues or cells [13]. Moreover, lncRNAs sequences are less conserved than mRNA among related species. Recently, secondary structures of lncRNAs have been shown to be conserved, having ‘repeat A’ region in lncRNA Xist (X inactive-specific transcript) and a ‘roX-boxes’ sequence motif comprised of two lncRNAs roX1 and roX2 [14, 15]. Research that lncRNAs have various important regulatory effect on target gene expression contributing to epigenetic modification, transcription and post-transcriptional processing via specific interactions with proteins and other cellular factors [16–18].

LncRNAs mediate epigenetic changes by recruiting chromatin remodeling complexes to specific genomic loci [16]. For example, the lncRNA HOX antisense intergenic RNA (HOTAIR)which is initiated from the HOXC cluster interacts with ploycomb repressive complex 2 (PRC2) to silence transcription across 40 kb of the HOXC locus in trans by inducing a repressive chromatin state [19]. lncRNAs, Xist, RepA and Kcnqot1 all recruit the polycomb complex to the target genome and they trimethylate lysine 27 residues (me3K27) of histone H3 to induce heterochromatin formation and repress gene expression [20, 21]. In addition, lncRNA also regulate target gene at transcriptional [18]. Proximal promoters can be transcribed into long ncRNAs that recruit and integrate RNA binding proteins function into the transcriptional process [22]. For example, an lncRNA induced by DNA damage and transcribed from the *cyclin D1* gene promoter, recruits and integrates RNA binding protein TLS to silence *cyclin D1* gene expression [22]. LncRNAs could act as co-factors to modulate transcription factor activity. LncRNA Evf-2 is transcribed from an enhancer and recruits transcription factor DlX2 to this same enhancer to induce expression of adjacent protein-coding genes [23]. Moreover, post-transcriptional regulation of lncRNAs is being revealed. Normally, lncRNAs are involved in splicing regulation and translational control [17]. The lncRNA MALAT1 (metastasis-associated long adenocarcinoma transcript 1) interacts with serine–arginine splicing factor to regulate its distribution in nuclear speckle domains and to modulate pre-mRNA alternative splicing [24]. A neuron-specific antisense lncRNA, AS Uchl1, could specifically induce the translation of ubiquitin carboxyl-terminal esterase L1(*Uchl1*) under certain stress conditions through its complementarity with target mRNA [25]. LncRNA brain cytoplasmic RNA 1 (BC1) blocks protein complex assembly to repress translation initiation in neurons and germ cells [26].

## Molecular archetypes of the lncRNA–protein interaction

Recently, how lncRNAs control gene expression and molecular function archetypes of lncRNAs have been concerned. Wang’s group discussed four emerging archetypes of molecular functions that lncRNAs execute as signals, decoys, guides, and scaffolds via proteins, DNA and RNA interaction [27]. Here, we distill the myriad functions of lncRNA into three archetypes of molecular mechanisms to illustrate how lncRNAs directly interacting with proteins and serve as ‘guide’ to recruit protein complexes to target genes, serve as ‘scaffold’ to assemble proteins into RNPs, and serve as ‘decoy’ to sequester regulatory proteins away from target gene [28]. We then offer examples of each archetype’s lncRNA–protein interactions.

### LncRNAs act as protein guides

**Fig. 1:**
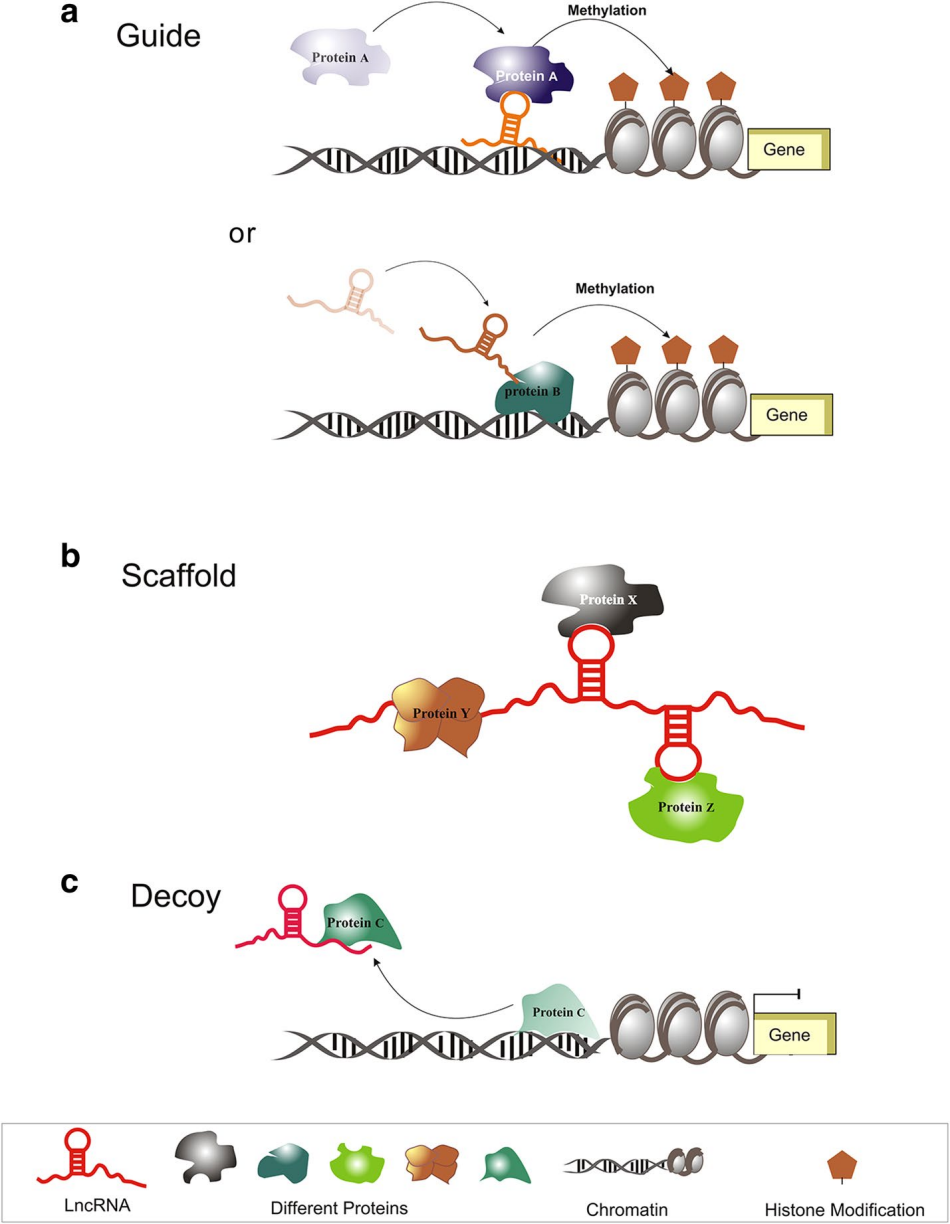
Schematic illustration of possible molecular archetypes of lncRNA–protein. **a** lncRNA guides protein to target genomic loci or the protein acts as an adaptor for lncRNA to link lncRNA to the target gene. **b** lncRNA brings two or three proteins together to form discrete complexes of lncRNA–RNPs. **c** lncRNA act as decoys to draw proteins away from t target genes

First, lncRNA acts as guide to recruit proteins to chromatin sites through RNA-DNA base pairing, to regulate downstream gene expression (Fig. 1a). For example, lncRNA fetal-lethal non-coding developmental regulatory RNA (Fendrr), which is specifically transcribed in nascent lateral plate mesoderm of the developing mouse embryo, guides PRC2 to target genes to increase PRC2 occupancy and trimethylate of H3K27me3, subsequently leading to attenuation of target gene expression [29]. Cold assisted intronic non-coding RNA (COLDAIR), a cold-induced lncRNA with a capped 5′ end lacking a 3′ poly A tail, is transcribed from the intron of the *FLC* locus gene in *Arabidopsis thaliana* and recruits PRC2 to establish and maintain stable repressive chromatin of *FLC* through H3K27 trimethylation during vernalization [30]. Other than recruiting PCR2 to repress target gene expression, some lncRNAs, such as lincRNA-p21 can guide hnRNP-K protein to the promoter of the *p21* gene and act as a co-activator for p53-dependent *p21* transcription [31]. Most known human proteins have nucleic acid binding domains [32], so potential lncRNA–protein interactions may act as adaptors to link lncRNA to target loci (Fig. 1a). The telomere complex is a classical model for proteins serving as adaptors between RNA and DNA [33, 34]. The telomere repeat factor TRF2 forms a stable complex with telomere-repeat-encoding RNA (TERRA) and telomere DNA repeats [35]. These interactions could be applied to lncRNA to illustrate archetypes for lncRNA and protein interactions. A well-studied protein that acts as an adaptor between regulatory lncRNA and chromatin is through YY1 tethering lncRNA of Xist to the inactive X nuclear center through repeat C. YY1 protein can bind both RNA and DNA through different sequence motifs and may serve as an adaptor for Xist [36].

### LncRNAs scaffolds bring proteins together

lncRNA can be scaffolds to create discrete protein complexes: lncRNA–RNPs (Fig. 1b). HOTAIR could both bind to PRC2 and LSD1 to repress gene transcription and the catalytic methyl-transferase subunit EZH2 of PRC2 is confirmed to be recruited via a structural domain at the 5′-end of HOTAIR to impart repressive histone modifications. Meanwhile the 3′-end of HOTAIR associates with LSD1, inducing H3K4 demethylated modification [37]. Another nascent antisense lncRNA, ANRIL, which is transcribed by RNA polymerase II at the TSS of the *p16*^*INK4a*^ gene, recruits PRC2 and PRC1 to mediate protein-coding gene repression *in cis* [38]. LncRNA roX is transcribed from the *Drosophila X* genome, which is thought to be critical scaffold for assembly of a functional MSL dosage compensation complex to activate transcription through acetylation of H4K16 [15, 39]. Moreover, lncRNAs act as protein scaffolds to control gene expression by modulation of nuclear architecture. The sub-nuclear structure-specific lncRNAs taurine upregulated gene 1 (TUG1) and nuclear-enriched autosomal transcript 2 (NEAT2)bind to methylated and unmethylated polycomb 2 protein (Pc2) respectively to mediate assembly of multiple corepressor or coactivator protein complexes [40], which switch non-histone protein methylated mark recognition to relocation of transcription units in the nuclear three-dimensional space, achieving coordinated gene expression regulation.

### LncRNAs act as decoys to titrate away proteins

lncRNAs act as decoys to remove proteins away from target loci (Fig. 1c). The lncRNA p21 associated ncRNA DNA damage activated (PANDA) is a well-studied lncRNA that acts as a decoy for transcription factors. PANDA is located 5 kb upstream of the *CDKN1A* with a 5′-cap and a 3′-polyadenylated but non-spliced tail. PANDA binds to and sequestrates NF-YA transcription factor from target gene promoters to repress gene expression [41]. Also, lncRNA could also be decoy of other proteins. LncRNA growth arrest-specific 5 (Gas5) has been identified to interact with glucocorticoid receptor (GR) to prevent binding to DNA response elements, thereby blocking glucocorticoid signal pathway [42]. The lncRNA metastasis-associated lung adenocarcinoma transcript 1 (MALAT1) binds to and sequesters several serine/arginine splicing factors to modulate their nuclear distribution and phosphorylation states to ensure splicing factor regulation of alternative splicing of cellular pre-mRNAs at a precise time, place, and concentration [24].

## Technologies to probe the lncRNA–protein interactions

AS the significance of lncRNA–protein interactions is better understood, their biochemistry attributes are being discovered and novel bioinformatics approaches are being developed to identify and predict proteins that interact with target lncRNA. Previously, most methods to study RNA–protein interactions are protein-centric, including native RNA immunoprecipitation (nRIP), cross-linking and immunoprecipitation (CLIP) [43, 44]. Recently, the discovery of numerous non-coding RNA has led to concern about RNA-centric approaches, such as the RNA pull-down assay, chromatin isolation by RNA purification (ChIRP), capture hybridization analysis of RNA targets (CHART), and RNA antisense purification (RAP) [45–48]. So other methods are being sought [49, 50], and here we depict detailed strategies for identifying lncRNA–protein interactions.

### Protein-centric approach to probing lncRNA–protein interactions

#### nRIP and nRIP-seq

nRIP is used to detect the association of individual proteins with specific RNA species in vivo. An antibody-based technology, this can be used to immunoprecipitate an RNA binding protein complex from a heterogeneous cellular homogenate in vivo. RNAs stably associated with the protein complex will immunoprecipitated together and associated RNAs including lncRNA and small RNA or even coding RNA can be measured with real-time PCR or be identified by RNA sequencing [51]. One important consideration prior to commencing RIP-based approaches designed to identify protein–RNA interactions is whether cross-linking is necessary. Native RIP is the method without crosslinking before cell lysis, which is a more appropriate for proteins that bind RNA directly. To reduce RNA recovery rates and identify the proteins that bind RNA indirectly, RIP assay is performed to crosslink cells with formaldehyde before cell lysis step [52]. nRIP is economical, requiring no specialized equipment and can be carried out under native conditions and thus allows identification of kinetically stable interactions [43]. However, the nRIP approach has limitations. First, differentiating direct from indirect interactions is difficult and. the read length of associated RNA is too large for identifying the actual binding site. Finally, nRIP assays are commonly known to have variability (reproducibility of 60 % or more) [53]. Thus, multiple biological replicates for nRIP are required. Thus, RIP-based approaches have been used to identify many lncRNA–protein interactions (see Table 1). Zhao’s group discovered that Xist, Tsix, and RepA interacted with PRC2 by native RIP [14]. Recently, Fendrr was identified to bind to PCR2 complex and WDR5 protein by nRIP [29]. Previously, RNA isolated by native RIP was analyzed with microarray technologies (RIP-ChIP) [54], but this is naturally limited by array design and coverage, so nRIP has recently been coupled with high-throughput sequencing (nRIP-seq) to capture the genome-wide RNA pools bound to target proteins [55]. Recent studies suggest that a genome-wide pool of thousands of PRC2-interacting RNAs including some characterized lncRNA, and many unannotated RNAs were identified in embryonic stem cells by nRNA-seq [56].

**Table 1:** Summary of lncRNA analyzed by several biochemistry approaches

LncRNAs	RBP	Biological function	Methods	Ref
ANRIL	PCR2, PCR1	Influences *p16* ^*INK4a*^ gene expression and cell senescence	CLIP	[38]
Air	G9a	Targets G9a in cis for imprinting	CLIP	[57]
Fendrr	PRC2, WDR5	Regulates gene in cis and in trans	RIP	[29]
FIRRE	hnPNPU	Modulation of nuclear architecture across chromosomes	RAP	[58]
HOTAIR	PCR2, LSD1	Silences transcription in trans via its modular architecture	RNA pull-down	[37]
Hottip	MLL-WDR5	Activates gene expression via chromosomal looping	RIP	[59]
lincRNA-p21	hnRNP-K	Mediates p53-dependent gene repression	RNA pull down-MS	[31]
MALAT1	PSPC1, PSF, PURA	Sequesters splicing factor to regulate alternative splicing	CHART-MS	[60]
NEAT1	PSPC1, SRSF1, ESRP2	Plays roles in RNA processing and transcriptional regulation	CHART-MS	[60]
Rox1	MLE, MSL	Mediates X chromosome upregulated to rescue male lethality	dChIRP	[61]
TERC	TCAB1	Functions as the template and scaffold for the telomerase complex	ChIRP	[47]
Xist	81 proteins (Hnrnpk, Spen)	Mediates chromatin modifications and Polycomb targeting	ChIRP-MS	[62]
Xist	10 proteins (SHARP, HDAC3)	Interacts directly with SHARP to silence transcription through HDAC3	RAP-MS	[63]

#### CLIP and CLIP-seq

CLIP is a powerful protein-centric tool used to isolate cross-linked RNA and protein complexes in tissues or cultured cells and subsequently purify the RNA targets. CLIP overcomes the drawbacks of nRIP by cross-linking RNA–protein complexes with ultraviolet light. Based on strong cross-links, followed by RNase treatment of the cell lysate to shorten RNA fragments, immunoprecipitation is performed to purify the covalently cross-linked lncRNA–protein. Importantly, covalent cross-linking permits stringent washing of immunoprecipitates, and this reduces background noise [44]. CLIP has been used to reveal that many intronic lncRNA directly bind to the PRC2 complex (see Table 1) [64]. For instance, Takashi’s group reported that lncRNA Air interacted with the H3K9 histone methyl-transferase G9a by CLIP [57], but their data suggest that the post-assay RNA quantity is small and the assay is tedious steps. Consequently, CLIP combined with high-throughput sequencing (HITS-CLIP or CLIP-seq) is used to identify many RBPs, such as Nova, Ago2, and TDP-43 [65–67]. The CLIP assay occasionally produces false–positive interactions and it determining the exact binding sites is not always straightforward [68]. Therefore, modified protocols have been developed to define cross-linking events, such as photoactivatable-ribonucleoside-enhanced CLIP (PAR-CLIP) and individual-nucleotide resolution CLIP (iCLIP) [69, 70]. With PAR-CLIP, cells are cultured in the presence of 4-SU (4-thiouridine) or 6-SG (6-thioguanosine), which are incorporated into RNA and induce strong cross-linking between RNAs and RBPs. Thus, this method can eliminate nonspecific targets and identify exact binding sites at a single nucleotide resolution [71]. Disadvantage of PAR-CLIP is difficulties in the use of 4-SU and 6-SG in living animals due to the toxicity. Thus, the PAR-CLIP method has been successfully applied to RBPs, such as HuR, FMRP, and Ataxin-2 [72–74]. To identify RNA binding motifs and novel functions, iCLIP introduces an adapter at the 5′ end through the primer used for reverse transcription by cDNA circularization and subsequent linearization. Thus, both truncated and read-through cDNAs are captured. Importantly, iCLIP also provides information about the cross-link site that permits precise mapping of RNA–protein contacts at a nucleotide resolution [70]. Rossbach’s group performed iCLIP combined with deep-sequencing (iCLIP-Seq) to reveal global regulatory roles of hnRPN L protein [75].

### RNA-centric approach to dissecting lncRNA–protein interactions

#### RNA pull-down assay

RNA pull-down assay is a preliminary RNA-centric in vitro method that enabling identification and characterization of various proteins which interact with a given lncRNA of interest. First, lncRNA probes were synthesized and labeled with high affinity tags, such as biotin, then cell lysate was prepared from a in vitro sample. Next, the lncRNA probe was incubated with lysate or recombinant protein to form a specific lncRNA–protein complex. Subsequently, the protein complex was pulled down with streptavidin agarose or magnetic beads. Finally, the retrieved protein was identified with Western blot or mass spectrometry (MS) [45]. Rinn’s group used RNA pull-down to discover that HOTAIR was directly associated with the PCR2 complex, which repressed transcription of the *HOXD* loci in trans [19].

#### ChIRP and ChIRP-MS

**Fig. 2:**
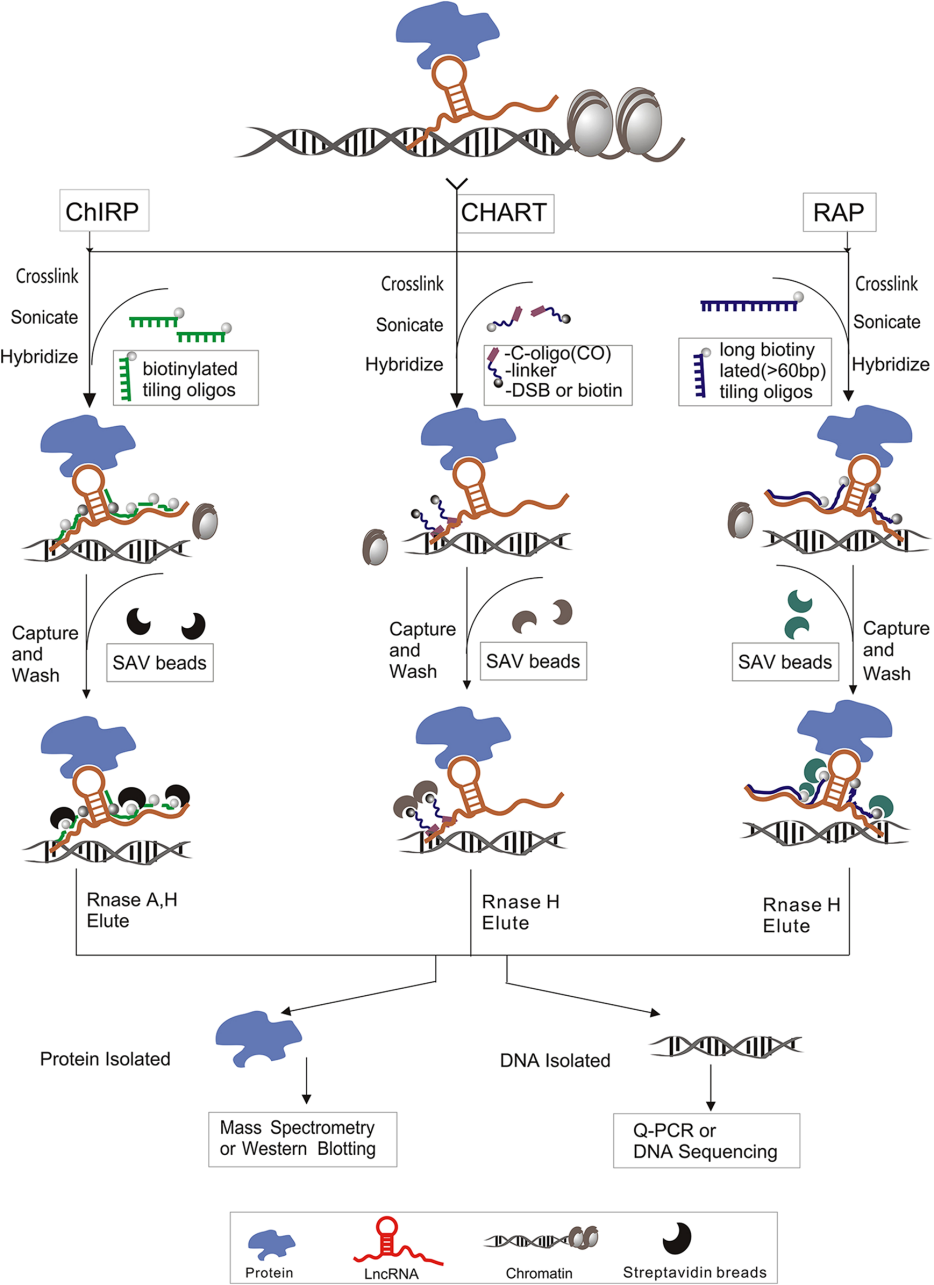
Schematic representation of ChIRP, CHART and RAP to identify associated proteins and chromatin DNA. RNA–protein–DNA complexes were cross-linked in vivo and solubilized by sonication. Corresponding biotinylated oligonucleotides of three methods were designed and synthesized to be hybridized to target lncRNAs under stringent conditions. Associated proteins and chromatin DNA were efficiently pulled down with streptavidin magnetic beads. Co-purified RNA, protein and DNA were isolated with RNase elution and subjected analyzed downstream analysis. Isolated proteins were analyzed by mass spectrometry or Western blotting and chromatin DNA was used for q-PCR, deep sequencing

Advances in RNA-centric biochemical purification offer new opportunities for systematically mapping lncRNA interactions with proteins and chromatin. The ChIRP method is based on using the biotinylated oligonucleotides complementary to the lncRNA of interest as a “handle” to pull down lncRNA-associated proteins and chromatin DNA. In brief, cultured cells are cross-linked, chromatin is extracted and sonicated, and then biotinylated tilling oligos that tile whole lncRNA were added and hybridized. Finally, the hybrids including target lncRNA, proteins and chromatin DNA were eluted with magnetic streptavidin beads and subjected to q-PCR or deep sequencing for DNA analysis, or to Western blotting or MS for protein analysis (Fig. 2) [46]. In addition, the recently developed ChIRP-seq method allows global and high-throughput discovery of genomic DNA associated with lncRNA. Chu’s group used ChIRP-seq to reveal the precision genomic occupancy of roX2, TERC, and HOTAIR: three rather different lncRNAs in two species successfully (see Table 1) [47]. To dissect the lncRNA functional domains in situ, domain-specific chromatin isolation by RNA purification (dChIRP) has been developed based on the ChIRP method and with dChIRP, biotinylated oligos are used as specific pools to recover specific domains of target RNA that improve the RNA genomic localization signal-to-noise ratio by >20-fold over traditional ChIRP [61]. dChIRP allowed lncRNA to be characterized at the domain level and revealed the lncRNA architecture and function with high precision and sensitivity [76]. Recently, Quinn’s group reported a ‘three-fingered hand’ topology of roX1 and that the three D domains of roX1 bind directly to the MSL protein complex to individually rescue male lethality by dChIRP [77].

MS based proteomics is a common tool for studying cellular interactions. Baltz and colleagues and Castello’s group used MS to identify hundreds of novel RBPs in human cells [78, 79]. Recently, to enable quantitation and accurate discovery of novel RNA–protein interactions from complexes assembled in vivo, Klass and colleagues used quantitative MS combined with RNase treatment of affinity-purified RNA–protein complexes to identify proteins that bind to RNA concurrently with an RBP of interest [80]. Kramer’s group developed an experimental and computational workflow method combining photo-induced cross-linking, high-resolution MS and automated analysis of the resulting spectra for identification of RNA interactions with proteins [81]. This MS-based workflow based on MS can be applied to map any RNA–protein complex of interest. Recently, ChIRP-MS, an optimized ChIRP method for systematically discovering lncRNA-bound proteome in vivo proteomes by MS was developed to identify 81 endogenous proteins that associated with Xist in two waves to coordinate X chromatin spreading and silencing. Interestingly, HrnpK protein participates in Xist-mediated gene silencing and chromatin modifications, but not Xist biogenesis or localization. Thus, the results suggested ChIRP-MS assay achieved high output and specificity regarding lncRNA–protein interactions in vivo [62].

#### CHART and CHART-MS

Another hybridization-based purification strategy is CHART which is used to confirmed the genome-wide localization of lncRNA in chromatin and isolate the protein associated with the lncRNA of interest. CHART is more similar than different with ChIRP (Fig. 2), but one significant difference is the design criteria of the oligonucleotide probe. With ChIRP, short antisense DNA oligonucleotides tile across the entire target lncRNA without a priori knowledge of target RNA function domain cover all potential hybridization spots [47]. In contrast, probes of CHART are empirically determined after RNase H assay which determines the candidate hybridization region [82]. CHART is a useful method for biochemically defining DNA and proteins associated with lncRNAs. CHART-seq, which combines CHART and RNA-seq, was applied to discover hundreds of trans-genomic binding sites for NEAT1 and MALAT1 [60]. Moreover, West’s group initially adapted the CHART assay to identify the full complement of proteins associated with RNAs in vivo with MS. CHART-MS was performed for two human lncRNAs, NEAT1 and MALAT1, to identify many nuclear speckle and para-speckle components and several new proteins not previously associated with them (see Table 1) [60].

#### RAP and RAP-MS

Similar to ChIRP and CHART, the RAP method is also used to capture a target lncRNA of interest through hybridization with antisense biotinylated oligos (Fig. 2) [47]. With RAP, various cross-linking conditions can be performed to identify different molecules that interact with the target RNA via different mechanisms. For direct RNA–RNA interactions, psoralens are used for cross-linking, but for protein–RNA interactions, formaldehyde or ultraviolet (UV) light is applied to crosslink. Compared to ChIRP and CHART, the most distinctive feature of RAP is its use of long capture probes (>60 nucleotides), which form very stable RNA-DNA hybrids [7]. Such a probe design strategy robustly captures any RNA and enables the use of stringent hybridization and washing conditions that dramatically reduce nonspecific interactions of off-target nucleic acids or proteins. Long DNA probes are considerably more costly but the background signals may be reduced due to the fewer probes used compared with short probes [48]. Hacisuleyman’s group applied RAP to discover the genomics sites and proteins that associated with lincRNA FIREE. And confirmed that it interacts with hnPNPU protein in an RRD-dependent manner and localizes across several trans-chromosomal binding sites (see Table 1) [58]. To developed a high-throughput method to identify proteins associated with a specific lncRNA in vivo, McHugh and colleagues combined RAP with MS to obtain high yields of RNA complex and identified ten proteins associated with lncRNA Xist, including SHARP, RBM15, MYEF2, CELF1, HNRNPC, LBR, SAF-A, RALY, HNRNPM, and PTBP1 (see Table 1). Also, they reported that the Xist interacts directly with SHARP to silence transcription through HDAC3 and that the recruitment of PCR2 by Xist depended on SHARP and HDAC3. These data is contrasted with previous work indicating that Xist directly interacted with PCR2 across the X chromosome [63]. Thus, the RAP-MS can be useful for investigating lncRNA regulation mechanism.

### Bioinformatics approach to predicting lncRNA–protein interactions

Biochemical approaches to identify the lncRNA–protein complexes are constantly expanding along with computational technologies. Compared with biochemical assays, the bioinformatics is more convenient and rapid for large-scale predictions of protein–lncRNA associations. Tartaglia’s group developed the algorithm, ‘fast predictions of RNA and protein interactions and domains at the Center for Genomic Regulation, Barcelona, Catalonia’ (catRAPID), which evaluates interaction propensities of polypeptide and nucleotide chains using their physicochemical properties [49]. catRAPID was used to predict RNA–protein interactions in neurodegenerative disorders, in which RNA-binding proteins apparently have a major role [83]. Recently, this method was used to predict protein interactions in the Xist regulatory network [84], and data show that catRAPID is powerful for predicting RNA–protein interactions from sequences. However, prediction of lncRNAs function is generally hampered by poor sequence homology and lack of interaction data. Consequently, Lu and colleagues developed a new computational method, lncPro, to predict lncRNA–protein interactions. Compared to CatRAPID, lncPro is computational-friendly and does not lead to nonsensical cross terms. Applying lncPro to all human proteins, this laboratory reported that long non-coding RNAs tend to interact with nuclear and RNA-binding proteins [50]. However, this technique is limited for finding the direct lncRNA–protein interaction s due to the volume of proteins. Recently, to gain insights into global relationships between lncRNAs and their binding proteins, Shang and colleagues constructed an lncRNA–protein network (LPN) including 177 lncRNAs, 92 proteins and confirmed 683 relationships between them, based on experimentally determined functional interactions [85]. Therefore, bioinformatics approaches to predicting lncRNA–protein interactions may guide future experimental approaches and facilitate a deeper understanding of the role of lncRNAs.

## Conclusions and perspectives

Given the multitude of non-coding transcripts discovered by second-generation deep sequencing, lncRNAs arouse interest to biological and bio-medical researchers. Recently, evidence has accumulated to support the idea that lncRNAs are critical to numerous biological processes, whereas the mechanisms by which lncRNA are poorly understood. Protein, an important partner for RNA in vivo, has been associated with molecular archetypes of lncRNA and we observed scaffolds, guides and decoys in these associations. However, some lncRNA interact with protein through more than one kind molecular mechanism. For example, HOTAIR is a scaffold for PCR2 and LSD1 as well as a guide to recruit PCR2 to target loci. Therefore molecular mechanisms behind lncRNA–protein interactions are complicated and rarely described. Study of lncRNAs interaction partners and the use of technologies to isolate and identify molecules associated with lncRNA are assisting researchers with the study of proteins and genomic DNA that directly and indirectly interplay with target lncRNAs. However, these interactions have not been studied across diverse species. As technologies improve, we may 1 day better understand evolution and functional mechanisms of lncRNAs.

## Abbreviations

lncRNA: long non-coding RNA
RBP: RNA binding protein
RIP: RNA immunoprecipitation
PRC2: ploycomb repressive complex 2
HOTTIP: HOXA transcript at the distal tip
HOTAIR: HOX antisense intergenic RNA
BC1: brain cytoplasmic RNA 1
Fendrr: fetal-lethal non-coding developmental regulatory RNA
COLDAIR: cold assisted intronic non-coding RNA
TUG1: taurine upregulated gene 1
NEAT2: nuclear-enriched autosomal transcript 2
Pc2: polycomb 2 protein
PANDA: p21 associated ncRNA DNA damage activated
Gas5: growth arrest-specific 5
MALAT1: metastasis-associated lung adenocarcinoma transcript 1
CLIP: cross-linking and immunoprecipitation
ChIRP: chromatin isolation by RNA purification
CHART: capture hybridization analysis of RNA targets
RAP: RNA antisense purification
PAR-CLIP: photoactivatable-ribonucleoside-enhanced CLIP
iCLIP: individual-nucleotide resolution CLIP
HITS-CLIP: high-throughput sequencing of CLIP
MS: mass spectrometry
LPN: lncRNA–protein network

## References

[1] Johnson JM, Edwards S, Shoemaker D, Schadt EE (2005). Dark matter in the genome: evidence of widespread transcription detected by microarray tiling experiments. Trends Genet.

[2] Trapnell C, Williams BA, Pertea G, Mortazavi A, Kwan G, Van Baren MJ, Salzberg SL, Wold BJ, Pachter L (2010). Transcript assembly and quantification by RNA-Seq reveals unannotated transcripts and isoform switching during cell differentiation. Nat Biotechnol.

[3] Djebali S, Davis CA, Merkel A, Dobin A, Lassmann T, Mortazavi A, Tanzer A, Lagarde J, Lin W, Schlesinger F (2012). Landscape of transcription in human cells. Nature.

[4] Novikova IV, Hennelly SP, Sanbonmats KY. Sizing up long non-coding RNAs: do lncRNAs have secondary and tertiary structure? Bioarchitecture. 2012;2:189–99. 10.4161/bioa.22592 PMC3527312 23267412

[5] Wilusz JE, Sunwoo H, Spector DL (2009). Long noncoding RNAs: functional surprises from the RNA world. Gene Dev.

[6] Yoon J, Abdelmohsen K, Gorospe M. Functional interactions among microRNAs and long noncoding RNAs. Seminars in cell and developmental biology. Elsevier; 2014. p. 9–14. 10.1016/j.semcdb.2014.05.015 PMC4163095 24965208

[7] Chu C, Spitale RC, Chang HY (2015). Technologies to probe functions and mechanisms of long noncoding RNAs. Nat Struct Mol Biol.

[8] Rinn JL, Ule J (2014). Oming in on RNA–protein interactions. Genome Biol.

[9] Buenrostro JD, Araya CL, Chircus LM, Layton CJ, Chang HY, Snyder MP, Greenleaf WJ (2014). Quantitative analysis of RNA–protein interactions on a massively parallel array reveals biophysical and evolutionary landscapes. Nat Biotechnol.

[10] Tome JM, Ozer A, Pagano JM, Gheba D, Schroth GP, Lis JT (2014). Comprehensive analysis of RNA–protein interactions by high-throughput sequencing-RNA affinity profiling. Nat Methods.

[11] Wang S, Tran E. Unexpected functions of lncRNAs in gene regulation. Commun Integr Biol. 2013; 6. 10.4161/cib.27610 PMC3917965 24563719

[12] Zhu B, Yang Y, Li R, Fu D, Wen L, Luo Y, Zhu H. RNA sequencing and functional analysis implicate the regulatory role of long non-coding RNAs in tomato fruit ripening. J Exp Bot. 2015;66:4483–95. 10.1093/jxb/erv203 PMC4507755 25948705

[13] Derrien T, Johnson R, Bussotti G, Tanzer A, Djebali S, Tilgner H, Guernec G, Martin D, Merkel A, Knowles DG (2012). The GENCODE v7 catalog of human long noncoding RNAs: analysis of their gene structure, evolution, and expression. Genome Res.

[14] Zhao J, Sun BK, Erwin JA, Song J, Lee JT (2008). Polycomb proteins targeted by a short repeat RNA to the mouse X chromosome. Science.

[15] Ilik IA, Quinn JJ, Georgiev P, Tavares-Cadete F, Maticzka D, Toscano S, Wan Y, Spitale RC, Luscombe N, Backofen R (2013). Tandem stem-loops in roX RNAs act together to mediate X chromosome dosage compensation in *Drosophila*. Mol Cell.

[16] Lee JT (2012). Epigenetic regulation by long noncoding RNAs. Science.

[17] Yoon J, Abdelmohsen K, Gorospe M (2013). Posttranscriptional gene regulation by long noncoding RNA. J Mol Biol.

[18] Ponting CP, Oliver PL, Reik W (2009). Evolution and functions of long noncoding RNAs. Cell.

[19] Rinn JL, Kertesz M, Wang JK, Squazzo SL, Xu X, Brugmann SA, Goodnough LH, Helms JA, Farnham PJ, Segal E (2007). Functional demarcation of active and silent chromatin domains in human HOX loci by noncoding RNAs. Cell.

[20] Terranova R, Yokobayashi S, Stadler MB, Otte AP, van Lohuizen M, Orkin SH, Peters AH (2008). Polycomb group proteins Ezh2 and Rnf2 direct genomic contraction and imprinted repression in early mouse embryos. Dev Cell.

[21] Pandey RR, Mondal T, Mohammad F, Enroth S, Redrup L, Komorowski J, Nagano T, Mancini-DiNardo D, Kanduri C (2008). Kcnq1ot1 antisense noncoding RNA mediates lineage-specific transcriptional silencing through chromatin-level regulation. Mol Cell.

[22] Wang X, Arai S, Song X, Reichart D, Du K, Pascual G, Tempst P, Rosenfeld MG, Glass CK, Kurokawa R (2008). Induced ncRNAs allosterically modify RNA-binding proteins in cis to inhibit transcription. Nature.

[23] Feng J, Bi C, Clark BS, Mady R, Shah P, Kohtz JD (2006). The Evf-2 noncoding RNA is transcribed from the Dlx-5/6 ultraconserved region and functions as a Dlx-2 transcriptional coactivator. Gene Dev.

[24] Tripathi V, Ellis JD, Shen Z, Song DY, Pan Q, Watt AT, Freier SM, Bennett CF, Sharma A, Bubulya PA (2010). The nuclear-retained noncoding RNA MALAT1 regulates alternative splicing by modulating SR splicing factor phosphorylation. Mol Cell.

[25] Carrieri C, Cimatti L, Biagioli M, Beugnet A, Zucchelli S, Fedele S, Pesce E, Ferrer I, Collavin L, Santoro C (2012). Long non-coding antisense RNA controls Uchl1 translation through an embedded SINEB2 repeat. Nature.

[26] Lin D, Pestova TV, Hellen CU, Tiedge H (2008). Translational control by a small RNA: dendritic BC1 RNA targets the eukaryotic initiation factor 4A helicase mechanism. Mol Cell Biol.

[27] Wang KC, Chang HY (2011). Molecular mechanisms of long noncoding RNAs. Mol Cell.

[28] Rinn JL, Chang HY. Genome regulation by long noncoding RNAs. Annu Rev Biochem. 2012;81:145–66. 10.1146/annurev-biochem-051410-092902 PMC3858397 22663078

[29] Grote P, Wittler L, Hendrix D, Koch F, Währisch S, Beisaw A, Macura K, Bläss G, Kellis M, Werber M (2013). The tissue-specific lncRNA Fendrr is an essential regulator of heart and body wall development in the mouse. Dev Cell.

[30] Heo JB, Sung S (2011). Vernalization-mediated epigenetic silencing by a long intronic noncoding RNA. Science.

[31] Huarte M, Guttman M, Feldser D, Garber M, Koziol MJ, Kenzelmann-Broz D, Khalil AM, Zuk O, Amit I, Rabani M (2010). A large intergenic noncoding RNA induced by p53 mediates global gene repression in the p53 response. Cell.

[32] Venter JC, Adams MD, Myers EW, Li PW, Mural RJ, Sutton GG, Smith HO, Yandell M, Evans CA, Holt RA (2001). The sequence of the human genome. Science.

[33] Zappulla DC, Cech TR. RNA as a flexible scaffold for proteins: yeast telomerase and beyond. Cold Spring Harbor symposia on quantitative biology. Cold Spring Harbor Laboratory Press; 2006. p. 217–24. 10.1101/sqb.2006.71.011 17381300

[34] Cech TR (2000). Life at the end of the chromosome: telomeres and telomerase. Angew Chem Int Ed.

[35] Bilaud T, Brun C, Ancelin K, Koering CE, Laroche T, Gilson E (1997). Telomeric localization of TRF2, a novel human telobox protein. Nat Genet.

[36] Jeon Y, Lee JT (2011). YY1 tethers Xist RNA to the inactive X nucleation center. Cell.

[37] Tsai M, Manor O, Wan Y, Mosammaparast N, Wang JK, Lan F, Shi Y, Segal E, Chang HY (2010). Long noncoding RNA as modular scaffold of histone modification complexes. Science.

[38] Wang H, Wang L, Erdjument-Bromage H, Vidal M, Tempst P, Jones RS, Zhang Y (2004). Role of histone H2A ubiquitination in Polycomb silencing. Nature.

[39] Maenner S, Müller M, Fröhlich J, Langer D, Becker PB (2013). ATP-dependent roX RNA remodeling by the helicase maleless enables specific association of MSL proteins. Mol Cell.

[40] Yang L, Lin C, Liu W, Zhang J, Ohgi KA, Grinstein JD, Dorrestein PC, Rosenfeld MG (2011). ncRNA-and Pc2 methylation-dependent gene relocation between nuclear structures mediates gene activation programs. Cell.

[41] Hung T, Wang Y, Lin MF, Koegel AK, Kotake Y, Grant GD, Horlings HM, Shah N, Umbricht C, Wang P (2011). Extensive and coordinated transcription of noncoding RNAs within cell-cycle promoters. Nat Genet.

[42] Kino T, Hurt DE, Ichijo T, Nader N, Chrousos GP (2010). Noncoding RNA Gas5 is a growth arrest and starvation-associated repressor of the glucocorticoid receptor. Sci Signal.

[43] Gong C, Popp MW, Maquat LE (2012). Biochemical analysis of long non-coding RNA-containing ribonucleoprotein complexes. Methods.

[44] Darnell R (2012). CLIP (cross-linking and immunoprecipitation) identification of RNAs bound by a specific protein. Cold Spring Harb Protoc.

[45] Feng Y, Hu X, Zhang Y, Zhang D, Li C, Zhang L. Methods for the Study of Long Noncoding RNA in Cancer Cell Signaling. Cancer Cell Signaling. Springer; 2014. p. 115–43. 10.1007/978-1-4939-0856-1_10 PMC4142042 24839023

[46] Chu C, Quinn J.Chang HY. Chromatin isolation by RNA purification (ChIRP). J Vis Exp. 2012;61. doi:10.3791/3912. 10.3791/3912 PMC3460573 22472705

[47] Chu C, Qu K, Zhong FL, Artandi SE, Chang HY (2011). Genomic maps of long noncoding RNA occupancy reveal principles of RNA–chromatin interactions. Mol Cell.

[48] Engreitz J, Lander ES, Guttman M. RNA antisense purification (RAP) for mapping rna interactions with chromatin. Nuclear bodies and noncoding RNAs. Springer; 2015. p. 183–97. 10.1007/978-1-4939-2253-6_11 25555582

[49] Bellucci M, Agostini F, Masin M, Tartaglia GG (2011). Predicting protein associations with long noncoding RNAs. Nat Methods.

[50] Lu Q, Ren S, Lu M, Zhang Y, Zhu D, Zhang X, Li T (2013). Computational prediction of associations between long non-coding RNAs and proteins. BMC Genom.

[51] Selth LA, Close P, Svejstrup JQ. Studying RNA–protein interactions in vivo by RNA immunoprecipitation. Epigenetics protocols. Springer; 2011. p. 253–64. 10.1007/978-1-61779-316-5_19 21913085

[52] Niranjanakumari S, Lasda E, Brazas R, Garcia-Blanco MA (2002). Reversible cross-linking combined with immunoprecipitation to study RNA–protein interactions in vivo. Methods.

[53] Khalil AM, Guttman M, Huarte M, Garber M, Raj A, Morales DR, Thomas K, Presser A, Bernstein BE, van Oudenaarden A (2009). Many human large intergenic noncoding RNAs associate with chromatin-modifying complexes and affect gene expression. Proc Natl Acad Sci.

[54] Keene JD, Komisarow JM, Friedersdorf MB (2006). RIP-Chip: the isolation and identification of mRNAs, microRNAs and protein components of ribonucleoprotein complexes from cell extracts. Nat Protoc Electron Ed.

[55] Cloonan N, Forrest AR, Kolle G, Gardiner BB, Faulkner GJ, Brown MK, Taylor DF, Steptoe AL, Wani S, Bethel G (2008). Stem cell transcriptome profiling via massive-scale mRNA sequencing. Nat Methods.

[56] Zhao J, Ohsumi TK, Kung JT, Ogawa Y, Grau DJ, Sarma K, Song JJ, Kingston RE, Borowsky M, Lee JT (2010). Genome-wide identification of polycomb-associated RNAs by RIP-seq. Mol Cell.

[57] Nagano T, Mitchell JA, Sanz LA, Pauler FM, Ferguson-Smith AC, Feil R, Fraser P (2008). The Air noncoding RNA epigenetically silences transcription by targeting G9a to chromatin. Science.

[58] Hacisuleyman E, Goff LA, Trapnell C, Williams A, Henao-Mejia J, Sun L, McClanahan P, Hendrickson DG, Sauvageau M, Kelley DR (2014). Topological organization of multichromosomal regions by the long intergenic noncoding RNA Firre. Nat Struct Mol Biol.

[59] Wang KC, Yang YW, Liu B, Sanyal A, Corces-Zimmerman R, Chen Y, Lajoie BR, Protacio A, Flynn RA, Gupta RA (2011). A long noncoding RNA maintains active chromatin to coordinate homeotic gene expression. Nature.

[60] West JA, Davis CP, Sunwoo H, Simon MD, Sadreyev RI, Wang PI, Tolstorukov MY, Kingston RE (2014). The long noncoding RNAs Neat1 and Malat1 bind active chromatin sites. Mol Cell.

[61] Quinn JJ, Chang HY. In situ dissection of RNA functional subunits by domain-specific chromatin isolation by RNA purification (dChIRP). Nuclear bodies and noncoding RNAs. Springer; 2015. p. 199–213. 10.1007/978-1-4939-2253-6_12 25555583

[62] Chu C, Zhang QC, Da Rocha ST, Flynn RA, Bharadwaj M, Calabrese JM, Magnuson T, Heard E, Chang HY (2015). Systematic discovery of Xist RNA binding proteins. Cell.

[63] McHugh CA, Chen C, Chow A, Surka CF, Tran C. The Xist lncRNA interacts directly with SHARP to silence transcription through HDAC3. Nature. 2015;521:232–6. 10.1038/nature14443 PMC4516396 25915022

[64] Ule J, Jensen K, Mele A, Darnell RB (2005). CLIP: a method for identifying protein–RNA interaction sites in living cells. Methods.

[65] Licatalosi DD, Mele A, Fak JJ, Ule J, Kayikci M, Chi SW, Clark TA, Schweitzer AC, Blume JE, Wang X (2008). HITS-CLIP yields genome-wide insights into brain alternative RNA processing. Nature.

[66] Chi SW, Zang JB, Mele A, Darnell RB (2009). Argonaute HITS-CLIP decodes microRNA–mRNA interaction maps. Nature.

[67] Lagier-Tourenne C, Polymenidou M, Hutt KR, Vu AQ, Baughn M, Huelga SC, Clutario KM, Ling S, Liang TY, Mazur C (2012). Divergent roles of ALS-linked proteins FUS/TLS and TDP-43 intersect in processing long pre-mRNAs. Nat Neurosci.

[68] Li Q, Uemura Y, Kawahara Y. Cross-linking and immunoprecipitation of nuclear RNA-binding proteins. nuclear bodies and noncoding RNAs. Springer; 2015. p. 247–63. 10.1007/978-1-4939-2253-6_15 25555586

[69] Hafner M, Landthaler M, Burger L, Khorshid M, Hausser J, Berninger P, Rothballer A, Ascano M, Jungkamp A, Munschauer M (2010). Transcriptome-wide identification of RNA-binding protein and microRNA target sites by PAR-CLIP. Cell.

[70] König J, Zarnack K, Rot G, Curk T, Kayikci M, Zupan B, Turner DJ, Luscombe NM, Ule J (2010). iCLIP reveals the function of hnRNP particles in splicing at individual nucleotide resolution. Nat Struct Mol Biol.

[71] Hafner M, Landthaler M, Burger L, Khorshid M, Hausser J, Berninger P, Rothballer A, Ascano M, Jungkamp A, Munschauer M. PAR-CliP-a method to identify transcriptome-wide the binding sites of RNA binding proteins. J Vis Exp. 2010;41. doi:10.3791/2034. 10.3791/2034 PMC3156069 20644507

[72] Ascano M, Mukherjee N, Bandaru P, Miller JB, Nusbaum JD, Corcoran DL, Langlois C, Munschauer M, Dewell S, Hafner M (2012). FMRP targets distinct mRNA sequence elements to regulate protein expression. Nature.

[73] Lebedeva S, Jens M, Theil K, Schwanhäusser B, Selbach M, Landthaler M, Rajewsky N (2011). Transcriptome-wide analysis of regulatory interactions of the RNA-binding protein HuR. Mol Cell.

[74] Yokoshi M, Li Q, Yamamoto M, Okada H, Suzuki Y, Kawahara Y (2014). Direct binding of Ataxin-2 to distinct elements in 3′ UTRs promotes mRNA stability and protein expression. Mol Cell.

[75] Rossbach O, Hung L, Khrameeva E, Schreiner S, König J, Curk T, Zupan B, Ule J, Gelfand MS, Bindereif A (2014). Crosslinking-immunoprecipitation (iCLIP) analysis reveals global regulatory roles of hnRNP L. RNA Biol.

[76] Lau E (2014). Non-coding RNA: zooming in on lncRNA functions. Nat Rev Genet.

[77] Quinn JJ, Ilik IA, Qu K, Georgiev P, Chu C, Akhtar A, Chang HY (2014). Revealing long noncoding RNA architecture and functions using domain-specific chromatin isolation by RNA purification. Nat Biotechnol.

[78] Baltz AG, Munschauer M, Schwanhäusser B, Vasile A, Murakawa Y, Schueler M, Youngs N, Penfold-Brown D, Drew K, Milek M (2012). The mRNA-bound proteome and its global occupancy profile on protein-coding transcripts. Mol Cell.

[79] Castello A, Fischer B, Eichelbaum K, Horos R, Beckmann BM, Strein C, Davey NE, Humphreys DT, Preiss T, Steinmetz LM (2012). Insights into RNA biology from an atlas of mammalian mRNA-binding proteins. Cell.

[80] Klass DM, Scheibe M, Butter F, Hogan GJ, Mann M, Brown PO (2013). Quantitative proteomic analysis reveals concurrent RNA–protein interactions and identifies new RNA-binding proteins in *Saccharomyces cerevisiae*. Genome Res.

[81] Kramer K, Sachsenberg T, Beckmann BM, Qamar S, Boon K, Hentze MW, Kohlbacher O, Urlaub H (2014). Photo-cross-linking and high-resolution mass spectrometry for assignment of RNA-binding sites in RNA-binding proteins. Nat Methods.

[82] Simon MD, Wang CI, Kharchenko PV, West JA, Chapman BA, Alekseyenko AA, Borowsky ML, Kuroda MI, Kingston RE (2011). The genomic binding sites of a noncoding RNA. Proc Natl Acad Sci.

[83] Cirillo D, Agostini F, Klus P, Marchese D, Rodriguez S, Bolognesi B, Tartaglia GG (2013). Neurodegenerative diseases: quantitative predictions of protein–RNA interactions. RNA.

[84] Agostini F, Cirillo D, Bolognesi B, Tartaglia GG. X-inactivation: quantitative predictions of protein interactions in the Xist network. Nucleic Acids Res. 2013;41:e31. 10.1093/nar/gks968 PMC3592426 23093590

[85] Shang D, Yang H, Xu Y, Yao Q, Zhou W, Shi X, Han J, Su F, Su B, Zhang C (2015). A global view of network of lncRNAs and their binding proteins. Mol Biosyst.

